# Perceptions of Quality of Care for Serious Illness at Different Levels of Facilities in a Rural Area of Bangladesh

**DOI:** 10.3329/jhpn.v27i3.3382

**Published:** 2009-06

**Authors:** Iqbal Anwar

**Affiliations:** Reproductive Health Unit, Public Health Sciences Division, ICDDR,B, GPO Box 128, Dhaka 1000, Bangladesh

**Keywords:** Healthcare, Healthcare-seeking behaviour, Health services, Patients' satisfaction, Quality of care, Bangladesh

## Abstract

This study was conducted to explore care-seeking for perceived serious morbidities and users' perceptions about quality of care at different facilities in Matlab, Bangladesh. This is a secondary analysis of baseline community survey data of the Matlab Essential Obstetric Care Project conducted in 2001. Principal component and factor analysis methods were used for computing summary quality and socioeconomic indicators. During perceived serious morbidity of any household member within the last one year, 88.1% (776/881) used health resource outside home. Of them, 25.6% visited informal care providers, 17.8% peripheral public facilities, 7.9% tertiary hospitals, 7.3% facilities of non-governmental organizations, and 41.4% private facilities as the highest healthcare resources. Socioeconomic status and type of morbidity were significant predictors for choice of the highest level of care. Most (86.1%) of those who sought care outside the home were satisfied with the quality of services provided for their last serious morbidities. Users of organized private-sector and tertiary facilities perceived the quality of services better than users of informal care providers and peripheral public facilities. Behaviour and attitude of the service providers and availability of medicines were significant predictors for perceived quality of care. Peripheral public-health facilities were of poor quality and grossly under-used. Further research should explore the technical aspect of quality of care in different facilities, along with perceptions of service providers to design client-focused interventions to impact the use of healthcare services. There is no reason to overlook informal care providers, they should rather be trained and monitored.

## INTRODUCTION

Bangladesh, like many developing countries, emphasized the development of government-owned healthcare establishments largely financed by tax-revenues. The country currently has a comprehensive government healthcare service-delivery system comprising peripheral primary healthcare centres and a tiered system of public hospitals that spans throughout the country. The rapidly-growing private sector is also increasingly contributing to the healthcare-delivery systems that include not-for-profit and for-profit private healthcare organizations and a cadre of informal service providers. With the establishment of such a system, an increasing attention was given on how to obtain greater impact from this service-capacity (1). The existing record shows that the use of peripheral government facilities is low (2), and this low use of government facilities is attributed mostly to poor quality of services (3,4).

Healthcare-seeking is a complex behavioural phenomenon. Literature suggests that the choice of care depends upon distance to facility, cost involved, and quality of care provided (5). However, care-seeking in presence of all these obstacles is also defined by illness-related factors, such as severity or nature of morbidity (6). Differential use of health services is also shaped by other factors, including socioeconomic status and gender (7,8). Results of one study suggest that socioeconomic status is not a barrier to the use when the population perceives that the benefit of service outweighs the cost (9).

Although quality is one of the most important determinants of service-use, it is not surprising that there is no consensus on how to define or measure quality. What emerges from the literature is its multi-dimensional nature (10-12). To define quality, experts used structure-process-outcome dimensions from the perspectives of patients, service providers, and managers (13). Understanding perceptions of populations about quality of care is critical to develop strategies to increase the use of health services (14,15). Quality-assessment studies usually measure one of three types of outcomes: medical outcomes, costs, or clients' satisfaction. For clients' satisfaction, service-recipients are asked to assess their satisfaction with the services delivered (16-18). With the passage of time, the experts realized that a single measure of general satisfaction is inadequate as an indicator of where, and how, any changes may be made to the service to satisfy patients and, indeed, to improve the real quality of services (technical). Nevertheless, studies have shown that the satisfaction of patients, although highly individualistic, depends, by and large, upon supply-side factors that relate to interpersonal skills of the service provider and the commodity elements in the setting where services are being provided (3).

Recent health policies in Bangladesh emphasize that limited public spending not only have an optimal impact on health at an affordable cost among people but also that health services are client-oriented (19). To avail of high-quality health services for all, it is essential to develop strategies that also cater to the poor. A better understanding of perceptions of population about quality of care should help develop strategies for equitable and sustainable health development of the country.

The paper aims to explore the patterns of use of the highest level of healthcare resources for perceived serious morbidities and the perceived quality of services provided in a rural area of Bangladesh.

## MATERIALS AND METHODS

This is a secondary analysis of baseline community survey data from the Matlab Essential Obstetric Care (EOC) Project, implemented in Matlab, Bangladesh, by ICDDR,B in collaboration with the Government of Bangladesh during 1998-2003. The community survey was conducted during December 2000–February 2001 for the evaluation of proposed safe motherhood interventions. A two-stage EPI (Expanded Programme on Immunization) 30 cluster-sampling procedure standardized by the World Health Organization (WHO) was used for identifying the study population. Villages were the primary sampling units (PSUs) while households were the secondary sampling units. Villages were selected with probability proportional to population size while households in selected villages were identified following the systematic sampling methodology. The household head was taken to be the respondent, and in his absence, the most informed and available adult member of the household—commonly the wife—was interviewed.

Trained interviewers were used for locating and interviewing household heads using structured questionnaire. The study focused on patterns of the use of healthcare resources in relation to the last episode of serious illness (as perceived by the household head or his proxy) of any family member during the previous one year and the perceived quality of services provided from different facilities. An ‘episode of serious illness' was defined as any perceived morbidity that warranted visit to a hospital or other care resources (although they might not have actually visited any facility). They were particularly asked about the highest level of health resources used, total household-level costs incurred, and the perceived quality of care provided. The cost of services in this study included total amount of money spent by the households as consultation/registration fees, bed-occupation fees, cost of transportation, price of medicines, cost of laboratory investigations, cost for improved diet, and others, such as cost of attendant, etc., to avail of services from that particular health facility or resources.

Quality-related questions covered six distinct areas: (a) behaviour and attitude of the service provider, (b) time spent for history-taking and physical examinations, (c) number of medicines prescribed and available from the facility, (d) satisfaction with levels of privacy maintained, (e) outcome of intervention, and (f) satisfaction with overall quality of care. Field supervisors and research investigators supervised data-collection activities to ensure a high degree of data quality.

Data were entered in the FoxPro database-management program and analyzed using the SPSS software (version 10). Using the principal component and factor analysis method, a composite socioeconomic status indicator (wealth index) was created using information on household assets, possession of land, construction materials for the main dwelling-house, source of drinking-water, type of latrine possessed, cash income, and expenditure of the household (20-22). Similarly, a composite quality indicator was computed from all quality-related variables, including behaviour and attitude of the service providers, time spent for history-taking and physical examinations, privacy maintained, availability of medicines, and outcome of treatment. One medical doctor classified the reported perceived serious illnesses into the following three groups of presumptive medical conditions: (a) acute minor, those likely to have been of a sudden onset and appeared minor in nature, like fever, diarrhoea, acute respiratory infection (ARI), etc.; (b) acute major, those likely to have been sudden in onset and grave in nature, like accidents, appendicitis, obstetric emergencies, etc.; and (c) chronic in nature, e.g. hypertension, diabetes, rheumatoid arthritis, etc.

Univariate and bivariate analyses were performed to understand the care-seeking patterns and quality of care at different health resources. Subsequently, a multivariate binary logistic regression model was built to look for the predictors of ‘care-seeking behaviour' and ‘perceived quality of care'.

## RESULTS

### Sociodemographic characteristics

In total, 2,177 households were included in the sample, and one adult respondent from each household was interviewed. Of the respondents, 638 (29.3%) were male, and 1,539 (70.7%) were female. The lower percentage of males is a reflection of day-time gender composition of households in rural Bangladesh when most men are in the fields for agricultural work. Most (89.9%) study subjects were Muslim, and 10.1% were Hindu. Of the male respondents, 80.7% were household heads while 66.0% of the females were spouses of household heads. The mean age of the respondents was 45.1 years for males and 35.9 years for females. The average household size was 5.5 members.

### Patterns of reported morbidities

**Fig. 1.**
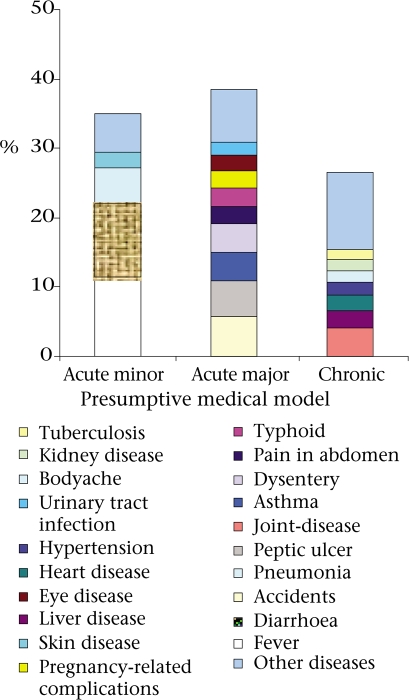
Distribution of the last reported seriousmorbidities within one year by presumptive medical model

During a one-year period preceding the survey, 99% (2,163/2,177) of the households in the study area reported at least one morbidity, and each household, on average, faced 4.3 morbid conditions in that period. More than 40% (881/2,177) of the households reported at least one member who had experienced an episode of illness perceived to be serious enough to warrant a visit to a hospital or a health facility (though not necessarily visited). The 10 most commonly-reported perceived serious morbidities were fever (11.6%), diarrhoea (10.4%), accidents and injuries (5.8%), pneumonia (5.1%), peptic ulcer (5.1%), joint-disease (4.2%), asthma (4.1%), dysentery (4.1%), pain in the abdomen (2.6%), and typhoid (2.6%). According to the ‘presumptive medical model', 35% were ‘acute minor' ailments, 38% were ‘acute grave' ailments, and the remaining 27% were chronic diseases (Fig. 1).

### Care-seeking patterns

For the last perceived serious morbidity, 88.1% (776/881) of the respondents visited health facilities or informal care providers outside the home, 9% (79/881) received treatment at home, and the remaining 2.9% (26/881) did not receive any treatment at all. Of those who visited a health facility or a service provider of some kind, 25.6% (199/776) consulted informal care providers as the highest health resources, who included unqualified, unregistered village doctors, medicine shopkeepers, homeopaths, *ayurvedic* practitioners, and traditional healers; 17.8% (138/776) visited primary- and secondary-level government facilities located at union, subdistrict, and district; 7.9% (57/776) visited tertiary government facilities, such as medical college hospitals and other specialized hospitals; 7.3% NGO facilities; and the remaining 41.4% (321/776) consulted organized private-sector care providers, including qualified practitioners and private clinics/hospitals, as the highest health resources (Table 1).

**Table 1.** Healthcare-seeking behaviour for perceived serious illnesses by sociodemographic characteristics of patients

Sociodemographic characteristics	Highest resources used for the last serious morbidities						
	Informal care providers	Government health facilities	NGO facilities	Organized private sector	Tertiary facilities	Total	p value
Socioeconomic status							
Poorest quintile	51 (39.2)	29 (22.3)	11 (8.5)	34 (26.2)	5 (3.8)	130 (100)	
2^nd^ quintile	47 (29.9)	31 (19.7)	15 (9.6)	59 (37.6)	5 (3.2)	157 (100)	
3^rd^ quintile	39 (27.9)	24 (17.1)	11 (7.9)	53 (37.9)	13 (9.3)	140 (100)	≤0.001
4^th^ quintile	35 (22.4)	28 (17.9)	08 (5.1)	69 (44.2)	16 (10.3)	156 (100)	
Richest quintile	27 (14.0)	26 (13.5)	12 (6.2)	106 (54.9)	22 (11.4)	193 (100)	
Sex of patients							
Male	106 (27.8)	70 (18.4)	28 (7.3)	144 (37.8)	33 (8.7)	381 (100)	0.35
Female	93 (23.5)	68 (17.2)	29 (7.3)	177 (44.8)	28 (7.1)	395 (100)		
Religion of patients								
Muslim	181 (25.8)	129 (18.4)	48 (6.8)	289 (41.2)	54 (7.7)	701 (100)	0.36
Hindu	18 (24.0)	9 (12.0)	9 (12.0)	32 (42.7)	7 (9.3)	75 (100)		
Age-group (years) of patients								
<5	48 (33.8)	28 (19.7)	30 (21.1)	31 (21.8)	5 (3.5)	142 (100)
5-14	37 (33.9)	20 (18.3)	12 (11.0)	34 (31.2)	6 (5.5)	109 (100)	
15-49	75 (20.8)	68 (18.8)	11 (03.0)	174 (48.2)	33 (9.1)	361 (100)	<0.001
50 and above	39 (23.8)	22 (13.4)	4 (02.4)	82 (50.0)	17 (10.4)	164 (100)	
Category of illnesses								
Acute minor	90 (33.0)	52 (19.0)	48 (17.6)	76 (27.8)	7 (2.6)	273 (100)	
Acute major	63 (20.8)	56 (18.5)	6 (2.0)	151 (49.8)	27 (8.9)	203 (100)	<0.001
Chronic	46 (23.0)	30 (15.0)	3 (1.5)	94 (47.0)	27 (13.5)	200 (100)	
Total	199 (25.6)	138 (17.8)	57 (7.3)	321 (41.4)	61 (7.9)	776 (100)	

Figures in parentheses indicate percentages; NGO=Non-governmental organization

In bivariate analysis (Table 1), use of the highest health resource for perceived serious morbidities varied significantly by socioeconomic status, type of perceived morbidity, and age-group of patients but not by sex or religion. Poorer families used informal services more than affluent ones (poor-rich ratio=2.8) while organized private and tertiary public facilities were used more by affluent respondents than poorer ones (poor-rich ratio 0.48 and 0.33 respectively). However, for use of NGO- and peripheral public facilities, the socioeconomic differentials were insignificant. Children aged less than 15 years used more NGO and informal care providers than their older counterparts while the older age-groups more used organized private-sector and tertiary public facilities. Thirty-three percent of the patients, suffering from any acute minor illnesses, visited informal care providers while patients suffering from acute grave and chronic morbidities more often visited the organized private sector. The NGO facilities were used mainly for acute minor ailments while the tertiary public facilities were used mainly for chronic diseases.

The estimated regression model showed that socioeconomic status and type of morbidity were significant predictors for care-seeking behaviour for perceived serious morbidities but not sex or religion of the patient. Patients from the richest socioeconomic quintiles were 73% (odds ratio [OR]=0.27; confidence interval [CI] 0.15-0.46) less likely to visit informal care providers than the poorest quintiles after controlling for the confounding effects of age, sex, religion of patients, and type of morbidity faced (Table 2). In multivariate analyses, age of the patient lost its significant association with the use of the highest health resources, although it showed significant relationship in bivariate analyses.

**Table 2.** Logistic regression analysis of use of informal care providers for treatment of perceived serious morbidities (1=Used informal providers; 0=Used other resources)

Independent variable	Reference categories	Odds ratio	95% CI for odds ratio
Socioeconomic status of households∗∗	Poorest quintile	1.00
	2nd	0.62	0.38-1.04
	3rd	0.61	0.36-1.04
	4th	0.44	0.26-0.76
	Richest	0.27	0.15-0.46
Age-group (years) of patients	<5	1.00
	5-14	1.06	0.61-1.83
	15-49	0.65	0.40-1.05
	50 and above	0.82	0.47-1.43
Illness category∗	Acute minor	1.00
	Acute major	0.65	0.43-0.98
	Chronic	0.85	0.53-1.36
Sex	Male	1.00
	Female	0.89	0.631-1.26
Religion	Mulsims	1.00
	Hindus	0.74	0.412-1.34

CI=Confidence interval

∗∗Highly significant

∗Significant

### Quality of care at different facilities as perceived by service recipients

Table 3 shows that users of health services were mostly positive about behaviour and attitude of the service providers. Thirty percent of organized private-sector users and 26% of tertiary facility users rated behaviour of care providers as ‘excellent' while the rates were 14% for users of informal health resources, 12% for users of Health and Family Welfare Centres (HFWCs), and 12.5% for users of Upazila Health Complexes (UHCs). Perception of users about attitude of the service providers was better when services were sought from private-sector facilities than from the government or NGO-sector facilities.

**Table 3.** Status of quality of care at different facilities as perceived by service users

Indicator	Facility availed								p value
	Informal care provider (n=199)	HFWC (n=49)	NGO facilities (n=57)	UHC (n=64)	District hospital (n=25)	Tertiary facilities (n=61)	Organized private sector (n=321)	Total (n=776)	
Behaviour of care providers (%)
Excellent	13.6	12.2	14.0	12.5	12.0	26.2	29.6	21.0
Good	84.4	71.4	80.7	73.4	76.0	60.7	64.4	72.8	<0.001
Average and below	2.0	16.4	5.3	14.1	12.0	13.1	6.0	6.2
Attitude of care providers (%)
Eager	69.3	42.9	52.6	54.7	36.0	55.7	73.5	64.8
Usual/normal	30.7	51.0	47.4	42.2	56.0	41.0	25.9	33.8	<0.001
Indifferent/annoying	0.0	6.1	0.0	3.1	8.0	3.3	1.6	1.4
Privacy maintained (%)	34.2	38.8	36.8	31.3	64.0	62.3	65.7	50.6	<0.001
Number of medicines prescribed (median)	3.0	3.0	2.0	4.0	4.0	4.0	4.0	4.0	<0.001
Number of medicines supplied (median)	1.0	1.0	2.0	0.0	1.0	0.0	0.0	0.0	<0.001
Time (minutes) spent for physical examinations (median)	10.0	10.0	7.0	10.0	15.0	20.0	15.0	10.0	<0.001
Opinion about overall quality of care (%)									
Excellent	9.5	10.2	26.3	4.7	8.0	16.4	23.4	16.6
Good	80.9	69.4	64.9	70.3	64.0	65.6	64.4	69.5	<0.001
Average and below	9.6	20.4	8.8	26.0	28.0	18.0	12.2	14.9	
Median distance covered (km)	1.0	1.0	3.0	3.7	20.0	40.0	16.0	4.0	<0.001
Median travel time (minutes)	30.0	30.0	50.0	60.0	180.0	120.0	300.0	60.0	<0.001
Total cost (Tk) (median)	370.0	350.0	100.0	859.0	3,880.0	3,400.0	2,000.0	900	<0.001

HFWC=Health and Family Welfare Centre; NGO=Non-governmental organization; UHC=Upazila Health Complex

With regard to privacy, 50.6% of the respondents stated that they were examined privately. According to perception of patients, privacy was well-maintained in the organized private-sector and tertiary facilities (district and above) than informal care providers, NGOs, and peripheral public facilities.

The median reported time for history-taking and physical examinations was 10 minutes that varied significantly by type of health facility (p<0.001). More time was spent when the patient visited tertiary facilities (20 minutes) or organized private facilities (15 minutes) than NGO facilities (7 minutes), informal private-sector care providers (10 minutes), or peripheral government facilities (10 minutes).

For the last reported morbidity, service-users reported prescription of four medicines (median) on average. However, this reported number of medicines prescribed varied significantly by type of morbidity and type of facility visited as the highest health resource. The availability of prescribed drugs, as reported by service recipients, was poor in the government facilities; only 19% of prescribed medicines were available from the government facilities while about 78% of prescribed medicines were available in the NGO facilities, and even the informal care providers supplied 54% of their prescribed medicines. Within the government facilities, there was a wide variation in the availability of prescribed medicines (Table 3).

The reported total household expenditure (median) for the treatment of the last episode of serious morbidity at the highest facility was Tk 900 (US$ 15) that included cost for transportation, attendants, and improved diets in addition to fees for consultation, bed occupation, and pathological tests. The household-level expenditure varied significantly by type of facility availed of and socioeconomic status. Patients for whom an NGO facility was the highest level of care, the reported median expenditure was Tk 100 only while it was Tk 370 when care was sought from informal care providers, Tk 350 for HFWCs, Tk 859 for UHCs, Tk 3,880 for district hospital, Tk 3,400 for tertiary facilities, and Tk 2,000 for organized private-sector facilities. Reported household-level costs at the higher-level government facilities were more than that of organized private-sector facilities.

The median distance between home and facility availed of for the last serious morbidity was four km. For accessing informal care providers and HFWCs, patients had to travel one km (median), for UHC 3.7 km, for organized private-sector facility 16 km, for district hospitals 20 km, and for tertiary facilities 40 km.

When asked about perception of the overall quality of services provided, 16.6% of the users mentioned that it was excellent, 69.5% considered quality as good, and the remaining 13.9% rated quality as average and below average. Those who visited the NGO facilities rated quality higher than those visiting another type of facility whereas those visiting the peripheral public facilities gave the lowest ratings. The significant predictors for the reported perceived quality of care, by order of strength of association were: (a) behaviour of service providers (p<0.001), (b) attitude of service providers (p=0.01), and (c) availability of medicines (p=0.05). The quality elements that could not predict the overall perceived quality were consultation time, level of privacy maintained, and total expenditure incurred (data not shown).

**Fig. 2.**
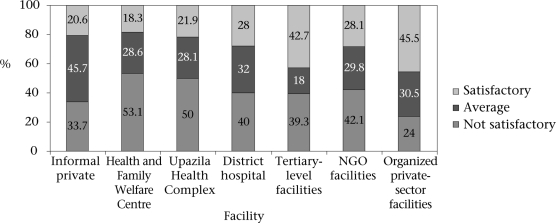
Distribution of users by their perceived quality of care in different health facilities

As mentioned in the methods, when a summary quality indicator was computed combining all quality variables and compared by facilities availed of, the results showed that the perceived quality varied significantly from facility to facility and in a consistent way (Fig. 2 and Table 4). Users of the organized private-sector facilities were 3.37 times more likely to be satisfied than users of informal care providers after controlling for the confounding effect of sociodemographic covariates and the severity/nature of the illness. Similarly, users of the tertiary facilities were three times more likely to be satisfied with quality of care than users of informal providers. Figure 2 and Table 4 show that the perceived quality of services from informal care providers is comparable with services from the peripheral government facilities. Among the public facilities, the perceived quality was better for the referral facilities, such as tertiary hospitals and district hospitals, than the peripheral UHCs and HFWCs. The overall perception about the quality of care provided did not vary by age, sex, religion, and socioeconomic status of the respondents but varied by nature of morbidity; those who suffered from acute minor diseases were more likely to be satisfied with the quality of care than patients with acute grave or chronic type of morbidities.

NGO=Non-governmental organization

**Table 4.** Logistic regression analysis for summary quality indicator (1=Satisfied; 0=Not satisfied)

Predictor/covariate	No. (776)	% satisfied with quality of care	Univariate model (odds ratio with 95% confidence interval)	Multivariate model∗ (odds ratio with 95% confidence interval)
Type of facility availed
Informal care providers	199	20.6	1.00 (reference category)	1.00 (reference category)
Government health facilities	138	21.7	1.07 (0.63-1.82)	1.06 (0.62-1.81)
NGO facilities	57	28.1	1.50 (0.77-2.95)	1.27 (0.63-2.56)
Tertiary facilities	61	42.7	2.86 (1.55-5.28)	3.01 (1.62-5.92)
Organized private sector	321	45.5	3.21 (2.14-4.83)	3.37 (2.18-5.22)
Category of illnesses (presumptive medical model)				
Acute minor	273	34.4	1.00 (reference category)	1.00 (reference category)
Acute major	203	32.3	0.91 (0.64-1.23)	0.61 (0.40-0.92)
Chronic	200	33.5	0.96 (0.65-1.41)	0.64 (0.40-1.01)

∗Controlled variables: All covariates in the model and age, sex, religion, and socioeconomic status

## DISCUSSION

Under-use of peripheral public facilities has been a major concern for the Government of Bangladesh despite the adequate geographical coverage throughout the country. The results of our study revealed that, during an attack of perceived serious morbidity of any kind, 41.4% of the patients visited organized private-sector facilities compared to 25.7% to the government facilities as the highest healthcare resources. A similar situation prevails in neighbouring India where results of a study of healthcare-seeking among the scheduled castes showed that 38% sought private medical help when their children became ill compared to only 28% from government health facilities (23). This is also apparent from the present study that, for more than one-fourth of rural populations even during their serious morbidities, the highest level of care was informal care providers.

One limitation of the study is that we considered the highest level of healthcare used for the last serious perceived morbidity. This may underestimate the use of lower-level facilities, including informal care provider as the patients often used several strategies/health resources to deal with symptoms before reaching the highest level of care. In addition, care-seeking behaviours for ailments that were not perceived as serious might be completely different.

The majority of the study patients were satisfied with the quality of services provided; this finding might reflect a low level of expectation owing to their life-long experience of spending a short time with healthcare providers. Other explanations for this high level of satisfaction with the quality of care could be that this was the highest level of care they sought for their last serious morbidity and was the preferred facility for seeking care. Our study depicts that perceptions of users about the quality of care varied significantly from facility to facility and by nature of disease (presumptive medical model) but not by age, sex, religion, or socioeconomic status of patients. Dissatisfaction with the quality of care has been present in a significant proportion of users of the peripheral government facilities and informal care providers. Qualitatively speaking, the perceived quality of care at the HFWCs and UHCs was comparable with the services of informal care providers. The service recipients from the organized private sector rated services to be the best and next to that were the tertiary facilities. Since the private care providers are not subsidized and depend on income from patients, they would be more motivated than the public hospitals to provide quality services to patients to meet their needs more effectively and efficiently. Others have reported similar results in this country (24,25). They have attributed the incentive structures under which they operate, as a key factor for this difference in the quality of care between public and private facilities.

Regarding the ‘perceived quality of care', the problem is that it may not reflect the actual quality of care (technical). Most rural population in Bangladesh are lay people; obviously, it is difficult for them to judge all technical aspects of quality of care. The findings of our study suggest that the lay people can perceive quality of services (26). The results of the present study also corroborate the findings of other studies that inter-personal communication, i.e. behaviour and attitude of service providers and the availability of medicines are significant predictors for better-perceived quality of care at the individual level but not consultation time or issue of maintaining privacy (3). To improve the perceived quality of services, interpersonal communication of service providers demands special attention, along with availability of essential drugs at the point of service-delivery.

According to the perspectives of users, quality services are available at the tertiary facilities and private-sector facilities that are located in urban areas and far from rural communities. Simultaneously, the reported costs of services from the organized private sector and central government facilities were systematically higher than services from the informal care providers, NGOs, or peripheral public facilities. The costs of treatment were more in the higher-level public facilities (district and above) than in the organized private-sector facilities, although government services are officially free. The possible explanations are that the majority who visited the district hospital or tertiary government facilities were admitted patients (27). Other explanations could be ‘hidden costs' in government hospitals that include ‘informal payments' to service providers and cost for buying medicines from outside markets (28,29). Higher cost of good-quality services and a longer distance between home and better-quality facilities certainly have a prohibiting effect upon their use, particularly by the poorest in the community. As a result, poorer households purchase poor-quality, ineffective and even dangerous services most frequently from small-scale, unregulated informal care providers. The results also suggest that ‘perceptions of quality' and better ‘ability to pay' drive wealthier patients to private facilities. The implication is that access cannot be equated simply with supply but is dependant on other demand-side factors (economic and social access) and, most importantly, upon the perceived quality of services. The results of our study confirm that the rapidly-growing private-sector facilities are playing a meaningful role in the provision of curative services in Bangladesh, justifying their existence, continuation, and growth. However, one study reported that they reduce quality by reducing inputs, disregard social pricing considerations or, worse, try to increase their profits by providing services that are unnecessary (30).

We found inequity in the use of the formal health sector in Bangladesh. Similar inequities have been reported recently from about half of the member countries of the Organization of Economic Cooperation and Development studied (31). Informal care providers are still dominant providers of curative care in rural areas of Bangladesh. Members from the poorest quintile households are more likely to visit an informal care provider than those from the richest quintile. It is undeniable, as has been shown in this study and others, that these informal care providers have been and will be the first choice for the great majority of the rural population, mostly the poor and other vulnerable groups. If we then agree that structural improvements are a requirement for a sustainable and equitable success in the health sector, which means a long process, the question “Would the institutionalization of these informal providers represent a feasible alternative for the health sector in terms of achieving goals?” deserves a serious consideration from policy-makers. Why not to increase their capacity and skills for delivering better services to the rural population where qualified practitioners are difficult to deploy? The activities of the informal group of healthcare providers should be monitored and brought under some regulatory mechanisms. The positive impact of this approach is likely to be seen in a relatively short time, and it overrides the operational, institutional or political constraints that addressing informal care providers may bring along. Within the existing policy-framework, a coherent approach is feasible.

In Bangladesh, the Government is committed to improving the quality of services as a means of increasing the use of government facilities. It is in this context of seeking a way to attract more people that the client focus emerges with singular force. Thus, it is necessary that increased efforts are to be oriented to clarify and specify the meaning, relevance, and limitations of the idea of ‘clients' satisfaction' or ‘perceived quality of care'. There has been a dearth of literature in this field. Further research should make more explicit which aspects of technical quality patients' satisfaction refer to; in other words, “to what extent does patients' satisfaction reflect the real level of quality of care (technical) received.” Hence, there is a need for careful exploration of the technical aspect of quality of all cadres of service providers (medical audit) to compare with the perceived quality of care as explored in this study. It is also imperative to understand “to what degree does the meaning of perceived quality differ between service users and service providers?” The results of such studies will enable policy-makers to design future interventions to improve the quality of care effectively, keeping a balance of care providers and clients' ideas of what quality of healthcare means.

## ACKNOWLEDGEMENTS

The Commission of the European Communities funded the study (Grant No. ALA/97/0038 and Contract No. BGD/B7-300/0038-01). ICDDR,B acknowledges with gratitude the commitment of the Commission of the European Communities to the Centre's research efforts.
